# Efficacy of Immediate Continuous Oxytocin Administration After Twin Cesarean Delivery

**DOI:** 10.7759/cureus.73952

**Published:** 2024-11-18

**Authors:** Akihito Morita, Daisuke Higeta, Ayuko Tanaka, Tatsuya Sato, Maki Inoue, Makoto Aoki, Akira Iwase

**Affiliations:** 1 Obstetrics and Gynecology, Gunma University Hospital, Maebashi, JPN; 2 Emergency, National Defense Medical College, Saitama, JPN

**Keywords:** cesarean section (cs), oxytocin regimen, propensity score-matching analysis, twin pregnancy, uterine atony

## Abstract

Objective: This study aims to investigate the efficacy of continuous oxytocin administration after completion of routine oxytocin administration during the third stage of labor in patients with twin pregnancies delivered via cesarean delivery (CD).

Methods: This retrospective case-control study was conducted between April 2014 and March 2024, and it included 156 women with twin pregnancies. The oxytocin group included patients who were administered continuous oxytocin 2 IU/h until 24 hours after delivery after completion of oxytocin 5 IU intravenous injection immediately after delivery as the routine procedure. The control group included patients who underwent the routine oxytocin procedure only. We conducted overlap propensity score-weighted analyses and inverse probability-weighted analyses in parallel to compare the incidence of postpartum hemorrhage (PPH) and adverse events within 24 hours after CD, including the mean amount of lochia, the incidence of blood transfusion, shock, and additional procedures to control bleeding, and reduction in hemoglobin (Hb) levels and hematocrit (Ht) values before and after CD.

Results: In the overlap propensity score-weighted analysis, the mean amount of lochia within 24 hours after CD, reduction in Hb levels, and reduction in Ht values were significantly lower in the oxytocin versus the control group (177 g and 383 g, respectively; (95% confidence interval (CI), -328.5 to -84.5); *P* < 0.01, 0.59 g/dL and 1.08 g/dL, respectively; (95% CI, -0.94 to -0.05); *P* = 0.03, and 1.75% and 3.37%, respectively; (95% CI, -2.94 to -0.29); *P* = 0.02, respectively). There was no significant difference in the total blood loss within 24 hours after CD and the incidence of any adverse events within 24 hours after CD, such as PPH, shock, additional procedures to control bleeding, and blood transfusion. Although the difference was not significant, the incidence of lochia exceeding 500 g was lower in the oxytocin versus the control group (4.8% and 26.4%, respectively; OR: 0.14 (95% CI, 0.02 to 1.17); *P *= 0.07). In the inverse probability-weighted analyses, the mean amount of lochia within 24 hours after CD was significantly lower in the oxytocin versus the control group (183 g and 387 g, respectively; (95% CI, -320.2 to -87.8); *P* < 0.01). Although there was no significant difference in the incidence of blood transfusion, the incidence of lochia exceeding 500 g or 1000 g was significantly lower in the oxytocin versus control group (4.6% and 27.2%, respectively; OR: 0.13 (95% CI, 0.04 to 0.41); *P *< 0.01, and 1.5% and 12.5%, respectively; OR: 0.10 (95% CI, 0.02 to 0.65); *P *< 0.01, respectively).

Conclusion: Continuous oxytocin administration after completion of oxytocin administration during the third stage of labor may reduce the incidence of PPH after CD for twin pregnancies.

## Introduction

Postpartum hemorrhage (PPH) contributes to increased maternal mortality and morbidity rates worldwide [1]. Twin pregnancy is an important risk factor for PPH. The incidence of PPH requiring blood transfusion is significantly higher in cesarean deliveries of twins versus singletons, with an incidence of 4.1% [2,3]. Approximately 80% of PPH cases are caused by uterine atony [4]. Several uterotonic agents, such as oxytocin, ergot alkaloids, and prostaglandins, are used to prevent PPH due to uterine atony. However, twin pregnancy is a high-risk factor for systemic diseases, including hypertensive disorders of pregnancy (HDP), and may discourage the use of various medications, including ergot alkaloids [5,6]. Therefore, oxytocin administration, which is less likely to be contraindicated in many cases, is primarily used during the third stage of labor to prevent PPH in twin deliveries. Hyperextension of the uterine myometrium is considered the main cause of the high incidence of uterine atony in twin pregnancies. To our knowledge, no reports have demonstrated decreased expression of oxytocin receptors in twin versus singleton pregnancies. Furthermore, the myometrium of the uterus reportedly contracts more in response to oxytocin in twin versus singleton deliveries, and oxytocin is reportedly a useful medication in twin deliveries as well [7].

Oxytocin is the most commonly used uterotonic agent, and while it has a rapid onset of action, it has a short duration of action [8-10]. Oxytocin is routinely administered as soon as a baby is delivered via cesarean section (CD). A postoperative intravenous oxytocin bolus (5-10 IU) is the routine prophylaxis. However, hospitals may decide whether to use a prophylactic or continuous oxytocin infusion after completion of the routine administration of oxytocin during the third stage of labor. Although several reports of oxytocin dosing protocols have been published, each examined singleton pregnancies, and prophylaxis for PPH in twin pregnancies has not yet been established [10,11].

This study aimed to investigate the efficacy of continuous oxytocin administration after completion of routine oxytocin administration during the third stage of labor in patients with twin pregnancies delivered via CD.

## Materials and methods

Study design

We conducted a single-center observational study of women pregnant with twins who delivered via CD at the perinatal center of Gunma University Hospital between April 2014 and March 2024. The study protocol was approved by the Institutional Review Board of Gunma University Hospital (approval number: HS2022-257). In our hospital, oxytocin (5 IU) in 100 mL of 0.9% NaCl solution is routinely injected intravenously immediately after the delivery of twins. The surgeon decides whether to use a continuous oxytocin infusion after completion of routine oxytocin administration during the third stage of labor based on their experience, the degree of uterine contractions, and the amount of bleeding up to that point. When used, oxytocin was administered at 2 IU/h until 24 hours after delivery. We did not administer rescue oxytocin other than that described above. In cases of massive bleeding, oxytocin was administered at the same dose as in the study protocol if oxytocin was not administered, and if oxytocin had already been administered, additional medication or another method of hemostasis was attempted.

In the present study, eligible patients were divided into two groups according to the oxytocin administration method. The women in the oxytocin group (n = 112) underwent continuous oxytocin administration after the routine procedure was completed. The women in the control group (n = 44) underwent only the routine procedure. Women in the control group were started on oxytocin if uterine contractions worsened after CD. To explore differences in maternal postpartum outcomes according to the oxytocin administration method, we collected baseline characteristics, primary clinical information, and biochemical data from their electronic medical records.

Study participants and outcomes

All patients with twin pregnancies during the study period were enrolled. Patients with placenta previa were excluded from the study. To evaluate maternal outcomes, the amount of lochia; incidence of blood transfusion within 24 hours after CD; shock (defined as shock index (SI, heart rate divided by systolic blood pressure) greater than one at any point) within 24 hours after CD; additional procedures to control bleeding (including intrauterine balloon tamponade or uterine artery embolization); and hemoglobin (Hb) levels and hematocrit (Ht) values before and after CD were obtained. SI has been proposed as a simple and quick method to assess the condition of a patient. The use of SI as one of the indicators of treatment may be important for the practice of early intervention, which is the most important factor in addressing PPH [12,13]. In Japan, PPH is defined as cumulative blood loss greater than 500 ml or more, whereas in some countries, it is defined as cumulative blood loss of 1000 ml or more. We presented results for both postpartum blood loss greater than 500 ml and greater than 1000 ml to allow for multifaceted utilization. This retrospective observational study included all cases except those meeting the exclusion criteria during the observation period. The ideal sample size was not calculated.

The study outcomes were the incidence of lochia exceeding 500 or 1000 g within 24 hours after CD, the mean amount of lochia within 24 hours after CD, the total blood loss within 24 hours after CD, that is, the sum of intraoperative blood loss and the amount of lochia within 24 hours after CD, blood transfusion within 24 hours after CD, shock within 24 hours after CD, additional procedures to control bleeding, estimated blood loss (EBL), and reduction in Hb levels and Ht values determined by maternal Hb levels and Ht values before versus after CD. EBL was calculated using preoperative and postoperative Ht and preoperative weight [14].

Statistical analysis

To clarify potential confounding factors in the association between prophylactic and continuous oxytocin administration and maternal morbidity, we conducted propensity score analysis using overlap weighting. Compared to the conventional propensity score method, an overlap weighting method can achieve an exact balance of the measured variables and optimize the accuracy of the estimated association between intervention and outcome. The generated target population mimicked the cohort of a randomized controlled trial without excluding patients from the original cohort [15]. A propensity score was created for each patient. Because an overlap weighting method has the disadvantage of reducing the sample size due to adjustments, an inverse probability weighting (IPW) was applied in parallel to estimate the effect of continuous oxytocin administered tentatively to all patients from the third stage of labor [16]. To create overlap weights for each patient, the propensity score was determined using a logistic regression model with prophylactic oxytocin as dependent variables and potential confounders, including maternal age, assisted reproductive technology (ART) pregnancy rate, multiparity rate, pre-pregnancy body mass index, Hb levels before CD, gestational age at delivery, the incidence of HDP, emergency CD rate, intraoperative blood loss, failure of uterine contractions immediately after fetal delivery, intraoperative infusion volume, and use or non-use of tocolysis within 24 hours before CD as the independent variables. Furthermore, to create inverse probability weights for each patient, the propensity score was determined using a logistic regression model with prophylactic oxytocin as dependent variables and potential confounders, including maternal age, ART pregnancy rate, multiparity rate, the incidence of HDP, intraoperative blood loss, failure of uterine contractions immediately after fetal delivery, intraoperative infusion volume, and use or non-use of tocolysis within 24 hours before CD as the independent variables. After the adjustment for these weights, a more balanced propensity-weighted sample was created with respect to the potential confounders included in the regression model. Group differences in baseline characteristics were compared using standardized mean differences (SMDs) [17]. We regarded SMDs of ≤ 0.1 as an acceptable balance of covariates between the groups. All tests were two-tailed, and P-values < 0.05 were considered statistically significant. The statistical analysis was performed using EZR version 1.52 (Saitama Medical Center, Jichi Medical University, Saitama, Japan), which is the graphical user interface for R version 4.02 (The R Foundation for Statistical Computing, Vienna, Austria) [18].

## Results

Baseline characteristics of the patients according to the oxytocin administration method (patients who received only routine oxytocin administration versus those who received continuous oxytocin administration after completion of routine oxytocin administration during the third stage of labor) are shown in Table 1. We performed propensity score overlap weighting to attain an adequate balance between the two groups. After overlap weighting, weighted background characteristics were balanced. After the IPW analysis, some variables had SMDs greater than 0.1, such as the incidence of HDP, failure of uterine contractions immediately after fetal delivery, and intraoperative infusion volume (Table 2).

**Table 1:** Characteristics of each group on the overlap weighting analysis Values are mean (SD) CD: cesarean delivery, BMI: body mass index, ART: assisted reproductive technology, SMD: standardized mean difference, Hb: hemoglobin level, SD: standard deviation

		Observed data			Overlap weighting	
	Oxytocin (n=112)	Control (n=44)	SMD	Oxytocin (n=23.2)	Control (n=23.2)	SMD
Maternal age (years)	33.6 (5.7)	34.0 (3.6)	0.11	33.8 (5.0)	33.8 (4.3)	< 0.001
Pre-pregnancy BMI	23.1 (4.9)	22.6 (6.7)	0.09	22.3 (4.8)	22.3 (6.4)	< 0.001
Multiparity, n (%)	52 (46.4)	33 (75.0)	0.61	14.7 (63.5)	14.7 (63.5)	< 0.001
ART pregnancy, n (%)	30 (26.8)	12 (27.3)	0.01	6.1 (26.4)	6.1 (26.4)	< 0.001
Hb before CD (g/dL)	10.8 (1.3)	10.6 (1.1)	0.10	10.6 (1.2)	10.6 (1.1)	< 0.001
Gestational age at delivery (weeks)	35.6 (4.0)	34.9 (3.4)	0.15	35.3 (3.7)	35.2 (3.3)	< 0.001
Hypertensive disorder of pregnancy, n (%)	25 (22.3)	13 (29.5)	0.17	5.3 (22.8)	5.3 (22.8)	< 0.001
Emergency CD, n (%)	57 (50.9)	28 (63.6)	0.26	1 (58.3)	14.3 (58.3)	< 0.001
Intraoperative blood loss (g)	1535 (620)	1429 (638)	0.17	13.8 (59.5)	13.8 (59.5)	< 0.001
Use of any tocolysis within 24 h before CD, n (%)	10 (8.9)	8 (18.2)	0.27	3.0 (12.9)	3.0 (12.9)	< 0.001
Abnormal uterine contraction during CD, n (%)	26 (23.2)	0.0 (0.0)	0.78	0.0 ( 0.0)	0.0 ( 0.0)	< 0.001
Intraoperative infusion volume (mL)	1488 (498)	1245 (338)	0.57	1332 (420)	1332 (308)	< 0.001

**Table 2:** Characteristics of each group on the IPW analysis Values are mean (SD). CD: cesarean deliver, BMI: body mass index, ART: assisted reproductive technology, SMD: standardized mean difference, IPW: inverse probability weighting, Hb: hemoglobin level, SD: standard deviation

		Observed data			IPW data	
	Oxytocin (n=112)	Control (n=44)	SMD	Oxytocin (n=110.4)	Control (n=35.9)	SMD
Maternal age (years)	33.6 (5.7)	34.0 (3.6)	0.11	33.6 (5.5)	33.8 (4.5)	0.04
Pre-pregnancy BMI	23.1 (4.9)	22.6 (6.7)	0.09	22.8 (5.0)	22.7 (7.5)	0.01
Multiparity, n (%)	52 (46.4)	33 (75.0)	0.61	59.1 (53.5)	19.0 (53.0)	0.07
ART pregnancy, n (%)	30 (26.8)	12 (27.3)	0.01	28.9 (26.2)	7.9 (21.9)	0.10
Hb before CD (g/dL)	10.8 (1.3)	10.6 (1.1)	0.10	10.6 (1.2)	10.7 (1.1)	0.05
Gestational age at delivery (weeks)	35.6 (4.0)	34.9 (3.4)	0.15	35.5 (3.9)	35.1 (3.5)	0.09
Hypertensive disorder of pregnancy, n (%)	25 (22.3)	13 (29.5)	0.17	24.9 (22.6)	10.2 (28.4)	0.14
Emergency CD, n (%)	57 (50.9)	28 (63.6)	0.26	61.4 (55.6)	20.5 (56.9)	0.03
Intraoperative blood loss (g)	1535 (620)	1429 (638)	0.17	1515 (616)	1437 (630)	0.13
Use of any tocolysis within 24 h before CD, n (%)	10 (8.9)	8 (18.2)	0.27	10.8 (9.8)	4.6 (12.9)	0.09
Abnormal uterine contraction during CD, n (%)	26 (23.2)	0.0 (0.0)	0.78	18.7 (16.9)	0.0 (0.0)	0.64
Intraoperative infusion volume (mL)	1488 (498)	1245 (338)	0.57	1425 (486)	1332 (302)	0.23

Overlap weighting analysis for the maternal clinical outcomes in each group is shown in Tables 3-4. Although there was no significant difference in the total blood loss within 24 hours after CD and EBL, the mean amount of lochia within 24 hours after CD was significantly lower in the oxytocin versus control group (177 g and 383 g, respectively; (95% confidence interval (CI), -328.5 to -84.5); P < 0.01). Reduction in Hb levels and Ht values were significantly lower in the oxytocin versus the control group (0.59 g/dL and 1.08 g/dL, respectively; (95% CI, -0.94 to -0.05); P = 0.03, and 1.75% and 3.37%, respectively; (95% CI, -2.94 to -0.29); P = 0.02, respectively). In addition, although the difference was not significant, the incidence of lochia exceeding 500 g was lower in the oxytocin versus the control group (4.8% and 26.4%, respectively; OR: 0.14 (95% CI, 0.02 to 1.17); P = 0.07). There was no significant intergroup difference in the incidence of lochia exceeding 1000 g, the incidence of shock within 24 hours after CD, the percentage of additional procedures used to control bleeding, and the incidence of transfusion at 24 hours after CD (P = 0.25, 0.28, 0.21, and 0.54, respectively).

**Table 3:** Overlap weighting analysis for maternal outcomes of continuous variables Values are mean (SD). CI: confidence interval, CD: cesarean delivery, Hb: hemoglobin level, Ht: hematocrit value, SD: standard deviation, EBL: estimated blood loss

	Oxytocin (n=23.2)	Control (n=23.2)	Estimate (β)	95% CI	P-value
Lochia 24 h after CD (g)	177 (223)	383 (505)	-206.5	-328.5 to -84.5	< 0.01
Total blood loss within 24 h after CD (g)	1637 (663)	1843 (946)	-206.5	-462.2 to 49.2	0.12
EBL (mL)	311 (647)	523 (777)	-212.9	-436.9 to 11.1	0.06
Reduction in Hb (g/dL)	0.59 (1.30)	1.08 (1.52)	-0.49	-0.94 to -0.05	0.03
Reduction in Ht (%)	1.75 (3.74)	3.37 (4.69)	-1.61	-2.94 to -0.29	0.02

**Table 4:** Overlap weighting analysis for maternal outcomes of binominal variables within 24 hours after CD CI: confidence interval, CD: cesarean delivery

	Oxytocin (n=23.2)	Control (n=23.2)	Odds ratio (95% CI)	P-value
Lochia > 500 g, n (%)	1.1 (4.8)	6.1 (26.4)	0.14 (0.02 to 1.17)	0.07
Lochia > 1000 g, n (%)	0.2 (0.7)	2.8 (12.2)	0.05 (0.00 to 8.41)	0.25
Incidence of postoperative blood transfusion, n (%)	0.2 (0.7)	0.8 (3.6)	0.19 (0.00 to 38.2)	0.54
Additional procedures to control bleeding, n (%)	0.9 (3.8)	3.6 (15.6)	0.21 (0.02 to 2.37)	0.21
Incidence of shock, n (%)	0.2 (0.8)	2.3 (9.8)	0.07 (0.00 to 8.58)	0.28

The IPW analysis for the maternal clinical outcomes in each group is shown in Tables 5-6. Although there was no significant difference in EBL, the total blood loss within 24 hours after CD, and the reduction in Hb levels and Ht values, the mean amount of lochia within 24 hours after CD was significantly lower in the oxytocin versus control group (183 g and 387 g, respectively; P < 0.01). The incidence of lochia exceeding 500 g and 1000 g within 24 hours after CD was significantly lower in the oxytocin versus the control group (4.6% and 27.2%, respectively; OR: 0.13 (95% CI, 0.04 to 0.41); P < 0.01, and 1.5% and 12.5%, respectively; OR: 0.10 (95% CI, 0.02 to 0.65); P = 0.02, respectively). In addition, the incidence of shock within 24 hours after CD (0.8% and 9.8%, respectively; OR: 0.07 (95% CI, 0.01 to 0.79); P = 0.03) and the percentage of additional procedures used to control bleeding (4.3% and 17.0%, respectively; OR: 0.22 (95% CI, 0.06 to 0.78); P = 0.06) were significantly lower in the oxytocin versus control group. There was no significant intergroup difference in the incidence of transfusion 24 hours after CD (P = 0.86).

**Table 5:** IPW analysis for maternal outcomes of continuous variables Values are mean (SD). CI: confidence interval, CD: cesarean delivery, Hb: hemoglobin level, Ht: hematocrit value, SD: standard deviations, EBL: estimated blood loss

	Oxytocin (n=110.4)	Control (n=35.9)	Estimate (β)	95% CI	P-value
Lochia 24 h after CD (g)	183 (233)	387 (499)	-204.0	-320.2 to -87.8	< 0.01
Total blood loss within 24 h after CD (g)	1699 (698)	1825 (914)	-126.1	-401.7 to 149.5	0.37
EBL (mL)	389 (648)	556 (774)	-167.1	-415.2 to 81.0	0.19
Reduction in Hb (g/dL)	0.72 (1.26)	1.15 (1.49)	-0.43	-0.92 to 0.05	0.08
Reduction in Ht (%)	2.19 (3.68)	3.56 (4.61)	-1.38	-2.81 to 0.05	0.06

**Table 6:** IPW analysis for maternal outcomes of binominal variables within 24 hours after CD CI: confidence interval, CD: cesarean delivery, IPW: inverse probability weighting

	Oxytocin (n=110.4)	Control (n=35.9)	Odds ratio (95% CI)	P-value
Lochia > 500 g, n (%)	5.0 (4.6)	9.8 (27.2)	0.13 (0.04 to 0.41)	< 0.01
Lochia > 1000 g, n (%)	1.6 (1.5)	4.5 (12.5)	0.10 (0.02 to 0.65)	0.02
Incidence of postoperative blood transfusion, n (%)	3.1 (2.8)	1.2 (3.3)	0.83 (0.10 to 7.11)	0.86
Additional procedures to control bleeding, n (%)	4.7 (4.3)	6.1 (17.0)	0.22 (0.06 to 0.78)	0.02
Incidence of shock, n (%)	0.9 (0.8)	3.5 (9.8)	0.07 (0.01 to 0.79)	0.03

## Discussion

We found that the continuous use of oxytocin immediately after delivery via CD in twin pregnancies decreased the mean amount of lochia within 24 hours after CD. Furthermore, continuous oxytocin administration was associated with a lower incidence of PPH, although the difference was not statistically significant. In twin pregnancies, continuous oxytocin administration after completion of oxytocin administration during the third stage of labor may be useful for the prevention of PPH.

Twin pregnancy is an important risk factor for PPH. A vaginal delivery trial is one strategy used to prevent PPH in twin pregnancies. However, not all twin pregnancies are suitable for vaginal delivery, and CD is often necessary due to maternal and fetal factors. Therefore, the present study focused on cases of CD. Because the myometrium in twin pregnancies is exposed to a higher degree of stretching due to the extra distension created by accommodating an extra fetus, PPH due to uterine atony is more likely to occur in twin pregnancies [3,19]. No statistically significant difference in the number of proliferating uterine myometrial cells was observed between singleton and twin pregnancies [20]. Significant differences in the contractile properties of the myometrium between singleton and twin pregnancies have been reported, with a significantly greater response to oxytocin in twin pregnancies [7].

Although there is no established prophylaxis for PPH in twin pregnancies, oxytocin is the most commonly used uterotonic agent. Identifying the optimal dose of oxytocin in patients undergoing CD is critical for reducing PPH and oxytocin-induced adverse events. There is much diversity in the usage of oxytocin due to the wide variety of regimens prescribed. Sheehan et al. reported that an intravenous bolus of oxytocin 5 IU and an additional infusion of oxytocin 40 IU did not change the incidence of massive bleeding but reduced the need for additional uterotonic agents [21]. An intravenous bolus of oxytocin 5-10 IU after delivery has been the standard prophylaxis, and whether to add a continuous oxytocin infusion to this regimen has been a point of contention. In many reports to date, PPH was reduced without increasing adverse events, although no difference in the amount of blood loss was noted [22]. These reports included multiple pregnancies as an exclusion criterion; therefore, the value of oxytocin use in multiple pregnancies remains controversial. In recent years, some reports have advocated the usefulness of carbetocin rather than oxytocin [23,24]. A study of the hemodynamic effects of oxytocin and carbetocin reported no significant differences between the two agents [25]. However, the efficacy and adverse effects of carbetocin remain unclear, and it has not been adopted in Japan.

The overlap weighting analysis in this study showed no significant difference in the rate of blood loss exceeding 500 g or 1000 g within 24 hours postoperatively and the incidence of any adverse events, including blood transfusion, postpartum shock, and additional procedures to control bleeding; however, the oxytocin group had less lochia within 24 hours postpartum and less reduction in Hb levels and Ht values. In contrast, the IPW analysis showed that the oxytocin group had a higher rate of lochia exceeding 500 or 1000 g within 24 hours postoperatively. Furthermore, the incidence of shock within 24 hours after CD and the percentage of additional procedures used to control bleeding were lower in the oxytocin group. In the present study, the results from the overlap weighting analysis were performed and presented as the main analysis because of the significant differences in background between the two groups. However, we also presented the results of the IPW analysis, since we believed that we should estimate the effect of continuous oxytocin administered tentatively to all patients from the third stage of labor. Thus, continuous oxytocin administration might be considered to reduce postpartum blood loss and the adverse events associated with PPH. Although several treatment methods are available for PPH, such as intrauterine balloon tamponade, compression sutures, and uterine artery embolization, there are few available methods for prophylaxis. There have been reports on the prophylactic use of compression sutures in twin pregnancies and the efficacy of tranexamic acid administration in singleton pregnancies [26,27]. Given the frequency of PPH in twin pregnancies, the invasiveness and adverse effects of these treatments, and their impact on subsequent pregnancies, these approaches are not yet practical. The use of these hemostatic devices is only one of the therapeutic strategies for PPH, and specific methods of oxytocin administration, which is the core prophylactic strategy for PPH in twin pregnancies, must be established.

This study has several limitations. First, the sample size is relatively small. After adjusting for overlap weights, a more balanced propensity-weighted sample was created with respect to the potential confounders. The IPW analysis was added because of the smaller sample size in the overlap weighting analysis. Second, since this study was limited to CD for twin pregnancies, it is unclear whether it can be applied to cases of vaginal delivery. Heesen et al. advocate changing the oxytocin dosage between intrapartum and scheduled CD because of the difference in sensitivity to oxytocin [11], but this grouping was not made in the present study. However, since there were few intrapartum CD cases in the present study, this point can be disregarded. Third, we could not rule out the possibility that oxytocin altered the maternal circulatory dynamics [28]. The presence or absence of signs of shock reflects the patient’s cardiovascular dynamics; however, since this information is highly subjective and some cases are not recorded in the medical record, we used the SI, which is objective.

## Conclusions

Our findings indicate that continuous oxytocin administration, particularly in CD for twin pregnancies, after completion of oxytocin administration during the third stage of labor may be considered to reduce the incidence of PPH. Multicenter studies are needed to establish specific methods for administering uterotonic agents during the postpartum period of twin pregnancies.
